# Evaluation of aging, diabetes mellitus, and skin wounds by scanning acoustic microscopy with protease digestion

**DOI:** 10.1080/20010001.2018.1516072

**Published:** 2018-09-06

**Authors:** Katsutoshi Miura, Kanna Yamashita

**Affiliations:** Department of Health Science, Pathology and Anatomy, Hamamatsu University School of Medicine, Hamamatsu, Japan

**Keywords:** Scanning acoustic microscopy, skin, aging, diabetes mellitus, wound, elasticity, protease

## Abstract

Scanning acoustic microscopy (SAM) can assess tissue stiffness by calculating the speed of sound (SOS) through tissues. SOS increases as tissue stiffness increases. Sensitivity to protease digestion depends on protein type, concentration, and modification. We analyzed the SOS images of formalin-fixed paraffin-embedded skin sections from elderly, young, diabetic, and nondiabetic subjects, as well as chronic and acute wounds. SAM provided high-resolution histology similar to LM and revealed characteristic SOS alteration following pepsin treatment. SOS values of dermis samples from elderly subjects (especially females) were lower than those of younger adults, which was indicative of age-related dermal softening and loosening. SOS values of elderly females were lower than those of younger females and elderly males. Dermal SOS showed a positive correlation with epidermal thickness. SOS values of epidermis of elderly subjects were higher than those of younger adults and showed a rapid decline 0.5h after protease digestion. Reticular dermis of diabetic patients exhibited greater pepsin resistance than that of nondiabetic patients. Chronic wounds exhibited greater SOS values and pepsin resistance than acute wounds. SOS variation with aging, diabetes mellitus, and wound fibrosis reflected histological and mechanical changes associated with senescence and disease duration. Epidermal thickness reflects age-related changes in dermal stiffness.

## Introduction

Increasing age and exposure to environmental factors are known to affect the physical properties of skin [1,2]. Skin consists of highly heterogeneous anisotropic materials and its composition and microscopic structure can vary as a consequence of aging and disease. Light microscopy (LM), in conjunction with special ancillary techniques (such as immunostaining), is useful for evaluation of both structural and biochemical changes during histological evaluation of biopsy specimens; however, assessment of mechanical properties that are relevant for diagnosis of several pathological conditions is not possible by LM. Therefore, in addition to the conventional histochemical methods, there is a need to develop micro-mechanical approaches that can measure the mechanical properties of discrete tissue components.

To understand the effect of histological changes on the mechanical properties of tissues, it is necessary to use techniques to measure elastic properties across usual thickness slides for routine histology [3]. Scanning acoustic microscopy (SAM) has evoked considerable interest as a method for assessment of both histological and elastic properties using the same section [4,5]. Atomic force microscopy-based nanoindentation is a candidate technique for mechanical testing [3,6]. Although this method offers higher resolution in length scale, identification of the observed material is difficult on light microscopic histology.

The speed of sound (SOS) through tissues obtained by SAM corresponds to soft-tissue elasticity and stiffness; therefore, harder tissues exhibit a greater SOS [7,8]. SOS values following protease digestion provide more information regarding tissue properties because they tend to vary based on the protein types, their density, and any structural changes, such as formation of cross-linkages [9].

SAM is superior to other observation methods. First, routine pathological sections are available not only as fresh and frozen but also as formalin-fixed, paraffin-embedded (FFPE) sections. SOS values of FFPE were shown to correlate with the elasticity of fresh organs and to remain stable after fixation for periods between 24 h and 3 months [9]. Second, images are available within a few minutes without staining. After observation with SAM, the same section may be used for light microscopic examination. Third, digital data of each point, such as SOS through various tissue elements such as blood, muscles, liver, and bone, are amenable to statistical analysis. Differences in SOS values are measurable between different lesions or during incubation in enzyme.

The most important application of SAM may be its ability to evaluate tissue stiffness. In the previous study, we used SAM with pepsin treatment to compare both the histological and mechanical alterations of several organs, such as aortic valves [10], myocardial infarcts [9], and mouse skin ulcers [9]. To apply SAM to skin tissue analysis, detecting skin stiffness is crucial for clinical diagnosis and treatments. We think aging, diabetes mellitus (DM), and wound healing show so variation in stiffness among lesions. The aim of this study is whether SAM can differentiate and evaluate these lesions.

## Materials and methods

### Materials and ethics

All human sections were obtained from the Hamamatsu University Hospital or the Shizuoka City Hospital archives. Skin biopsy specimens of facial skin were selected to examine age-related changes and DM-induced damage whereas skin from the lower leg was used for the wound lesions. FFPE blocks were flat-sectioned into 10-µm thick slices and observed by SAM. The research protocol for use of stored samples with no link to patient identity was approved by the Ethics Committee of Hamamatsu University School of Medicine (No. 14-135). The requirement for written consent of patients was waived off in this retrospective study. The methods were carried out according to the approved guidelines and regulations.

### Grouping by ages, presence or absence of DM and acute or chronic wounds

For evaluation of age-related changes in skin, the skin samples were categorized into three groups based on the age of patients: (1) Group A: younger adults aged 20–50 years (n = 6; four males and two females); (2) Group B: older adults aged 51–74 years (n = 6; three males and three females); and (3) Group C: elderly individuals aged ≥75 years (n = 6; three males and three females).

For evaluation of skin changes associated with DM, we compared facial specimens of patients with and without a history of DM [DM skin samples: n = 5; four males and one female; average age (±standard deviation): 71.4 (±11.2) years; non-DM skin samples: n = 11; six males and five females, average age: 80.7 (±18.2) years].

To evaluate skin changes associated with acute and chronic wounds, we selected skin excisional biopsies with a history of foot ulcers. Wounds with granulation tissue and inflammatory cells were considered to be acute wounds, and those with fibrous scars with rich collagen fibers and scarce inflammatory cells were regarded as chronic wounds [acute wound specimens: n = 5; average age: 58.4 (±9.8) years; chronic wound specimens: n = 4; average age: 58.3 (±18.2) years].

### SAM observations

The Scanning acoustic microscope (AMS-50AI) was manufactured by Honda Electronics (Toyohashi, Aichi, Japan) and was equipped with a 320-MHz transducer, which had a resolution of approximately 4.7 µm. The SAM used ultrasound to image an object by plotting the speed or attenuation of sound through the sections on the screen (supplemental figures). As harder tissues result in greater SOS values through the sections, SAM can provide data on tissue elasticity and lesions [4,8].

The following characteristics were compared for assessment of age-related changes: reticular dermis composed of thick collagen fibers; papillary dermis comprised of scanty collagen and elastic fibers; and prickle cell layer of the epidermis. Facial skin biopsy specimens were chosen for this purpose as facial skin is more vulnerable to age-related damage owing to greater exposure to sun.

For comparison between DM and non-DM skin, SOS values of the facial skin reticular dermis were evaluated. We hypothesized that glycation cross-linking increases with DM. To test this hypothesis, collagen in reticular dermis is a good target to evaluate these modification because turnover of collagen is higher than that of other proteins and the receptors for advanced glycation end products (AGEs), which are vulnerable to DM changes [11], are abundantly expressed in this portion.

For examination of skin wounds, the areas in close proximity to granulation tissues were designated as acute wounds, whereas older scar regions were considered as chronic wounds.

### Protease digestion

Paraffin sections were dewaxed in xylene, soaked in distilled water, and submerged in a pepsin (Sigma P-6887) solution (3 units/mL in 10 mM HCl) at 37°C for 0.5 and 2 h [9]. The sections were washed in distilled water and observed under SAM. The same sections were used for repeated digestion.

### Light microscopic observation

To compare SAM with LM images, the same or nearby sections were stained with haematoxylin and eosin and Elastic van Gieson (EVG) stain. On EVG staining, the collagen and elastic fibers were stained red and black, respectively.

The magnification of the images was calibrated to those of SAM images, the scales for which are shown on the basal horizontal bars on the screen.

### Epidermal thickness

Epidermal thickness was measured on HE-stained slides. Distance from the basement membrane to the top of the prickle cell layer beneath the horny layer was calculated. Segments of epidermis between rete ridges were measured at least 5 times and the average thickness is reported.

### Graphs of SOS values and statistical analysis

The SOS values of at least five different points randomly chosen from each image were collected and statistically analyzed. For comparisons, the average (±SD) SOS values for each group are reported. For age-related alteration of SOS, average SOS values were plotted according to each age. For comparison of gender difference, the data points for males and females are indicated in different colors. To examine the correlation between epidermal thickness and dermal SOS, average SOS of reticular and papillary dermis were plotted according to each epidermal thickness to make a regression line.

To follow SOS values after pepsin treatment, both genders were included in each group. The average values were compared at 0 h, 0.5 h, and 2 h by paired *t* test, with *P* < 0.05 considered statistically significant. Before conducting the statistical analyses, we confirmed the normal distribution using a test for the difference between averages.

One-way analysis of variance was used to compare SOS values among different groups of age and gender. Multiple comparisons were assessed using Tukey–Kramer’s test, with *P* < 0.05 considered statistically significant.

## Results

### Differences in SOS imaging associated with skin aging and gender

**Figure 1.**
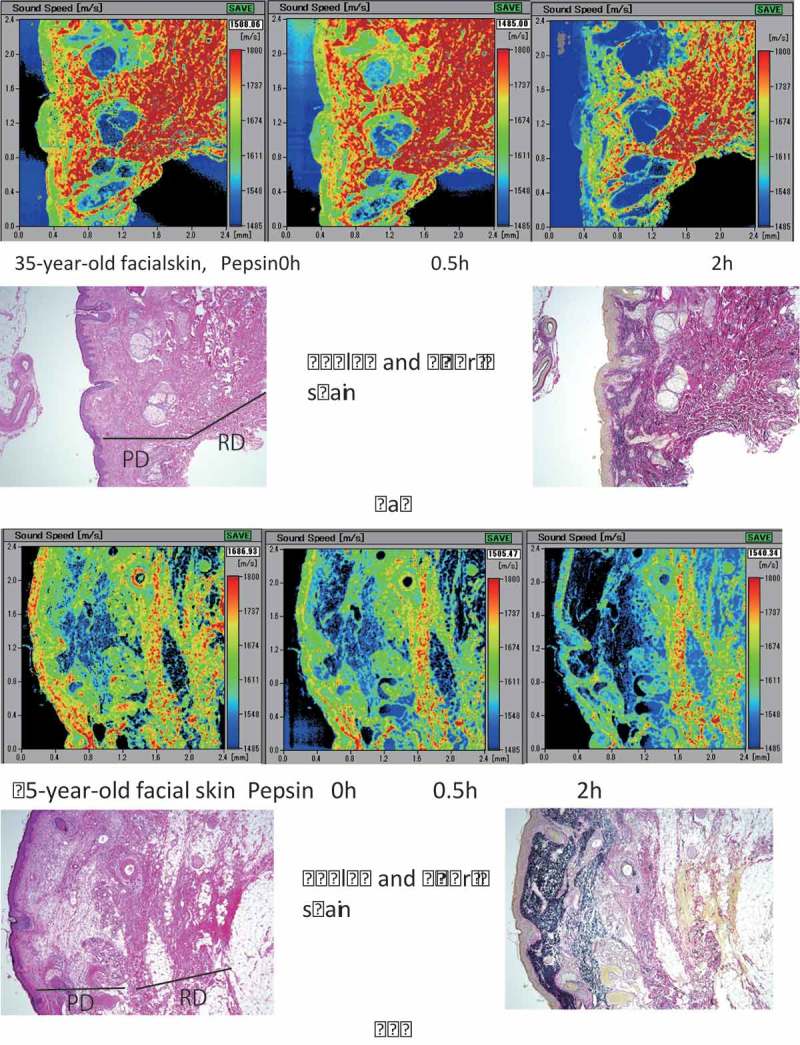
Speed of sound (SOS) images of younger adult (a) and elderly (b) skin samples with pepsin digestion. (a) Prior to pepsin digestion, higher SOS values were observed in the reticular and papillary dermis, whereas lower values were associated with the epidermis. Following pepsin digestion, SOS values of both dermis and epidermis gradually decreased. Dermis composed of thick collagen maintained high SOS values even after 2 h digestion. (b) Dermis from the elderly had lower SOS values than that from younger adults. SOS values in the epidermis were higher in the elderly than in young adults. After digestion, SOS values of both the epidermis and dermis gradually decreased, especially the papillary dermis and epidermis. PD, papillary dermis; RD, reticular dermis.

SAM provided high-resolution histology comparable to LM and revealed the SOS reduction following pepsin treatment. SOS skin images of younger adults (Figure 1(a)) showed a greater number of regions comprised of thick bundles in the reticular and papillary dermis, which corresponded to a dense collagen network. In contrast, elderly skin samples (Figure 1(b)) showed lower-SOS areas composed of more fragmented or fine fibers as compared to those of younger adults; this reflected age-related diminution in thick collagen bundles. In addition, the papillary dermis exhibited low SOS values, which corresponded to degenerated collection of elastic fibers associated with collagen loss.

**Figure 2.**
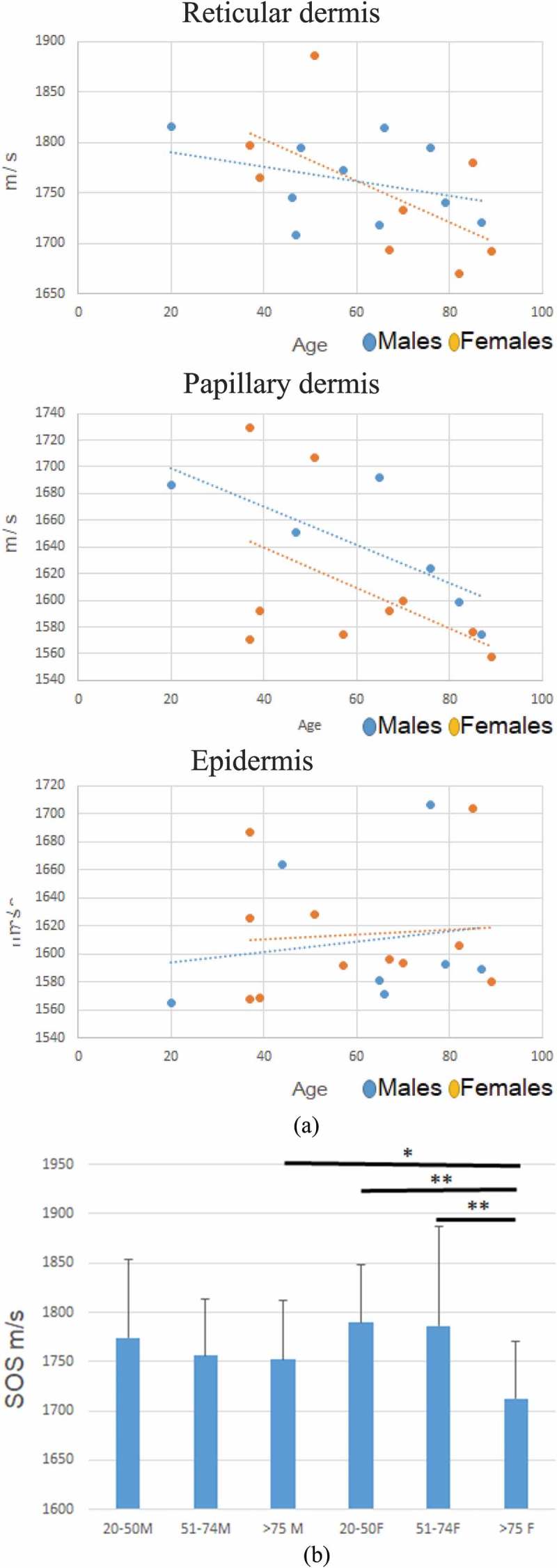
Age-related differences in SOS between males and females. (a) Average SOS of reticular dermis (top), papillary dermis (middle), and epidermis (bottom) were plotted according to each age. SOS of reticular and papillary dermis of both males and females decreased with age while SOSs of epidermis slightly increased with age. Between males and females, SOS of reticular dermis of females rapidly declined compared with males. (b) SOS of reticular dermis of males and females among different age groups were compared. Between males and females, only elderly individuals showed significant difference (*P *= 0.016). In females, elderly individuals (>75) showed rapid reduction of SOS compared with older adults (51–74) (*P *= 0.0026). All SOS values are presented as mean ± standard deviation.

Figure 2(a) shows age-related differences in SOS between males and females. SOS of reticular dermis and papillary dermis significantly decreased with age while that of epidermis slightly increased with age. A significant difference was observed between males and females ≥75 years of age with respect to SOS of reticular dermis (*p *= 0.016) (Figure 2(b)). SOS of reticular dermis of females aged ≥75 years was significantly lower than that of females in the 51–74 years age-group (*P *= 0.0026). However, no significant difference in this respect was observed between different age-groups of males.

### Age-related differences in SOS values following pepsin treatment

**Figure 3.**
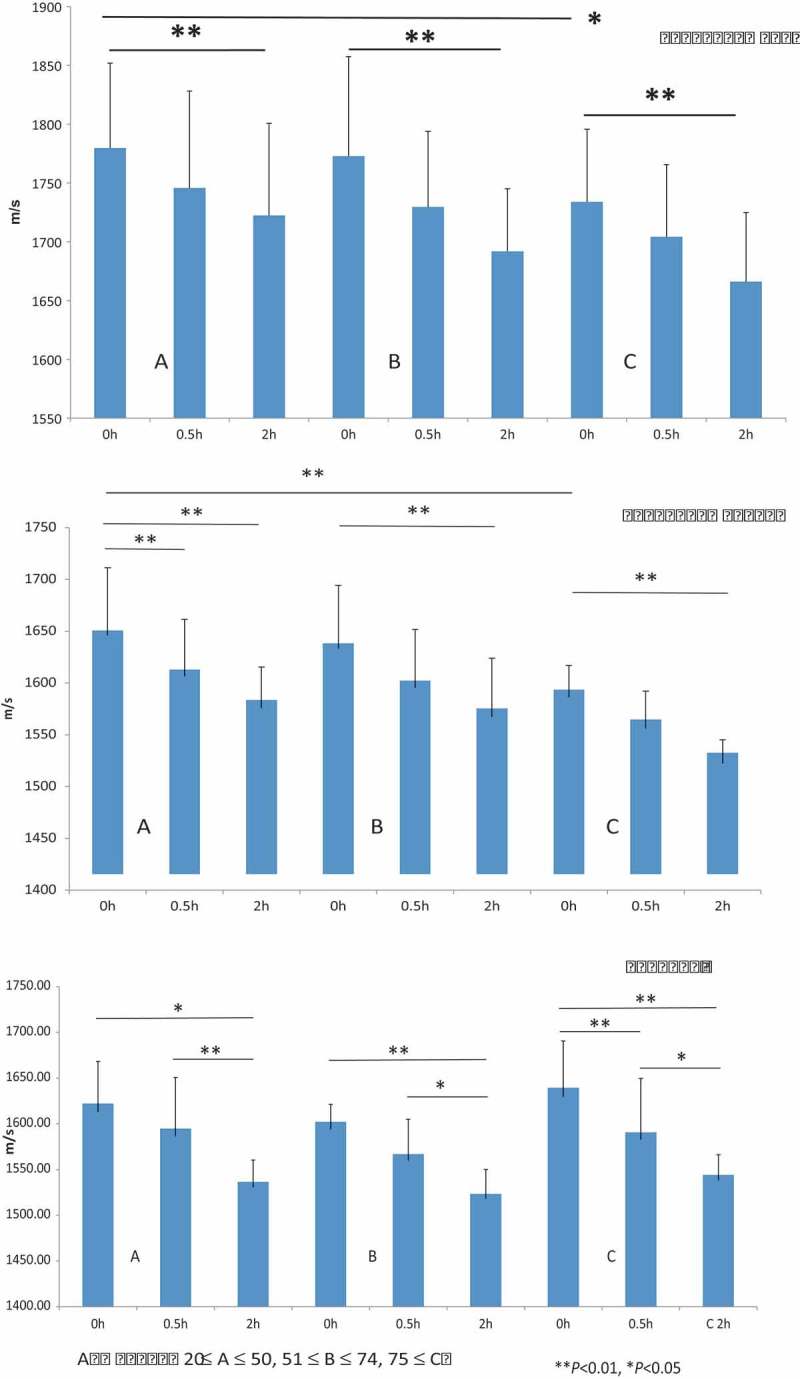
SOS after pepsin digestion was compared among the various age groups. (Top) The reticular dermis of the younger adults presented significantly greater SOS values than that of the elderly samples (*P *< 0.05). Pepsin digestion induced a significant decrease in SOS values for each age group after 2 h (*P *< 0.01). (Middle) The papillary dermis of the younger adults presented significantly greater SOS values than those of elderly individuals (*P *< 0.01). After pepsin digestion, there was a significant reduction in SOS values in each age group after 2 h (*P *< 0.01). (Bottom) The epidermis of elderly individuals had higher SOS values as compared to that in younger and older adults. After digestion, the epidermis in the elderly skin samples experienced a significantly rapid reduction in SOS values at 0.5 h (*P *< 0.01). The SOS values of the younger and older adults were also significantly decreased after 2 h. All SOS values are presented as mean ± standard deviation. Age group: 20 ≤ A ≤ 50, 51 ≤ B ≤ 74, 75 ≤ C; **P < 0.01, *P < 0.05, Tukey–Kramer test.

Figure 3 and Table 1 present the dermal SOS values following pepsin treatment among the younger adults (Group A), the older adults (Group B), and the elderly (Group C). The SOS values of reticular (Figure 3 *top*) and papillary (Figure 3 *middle*) dermis in Group A were significantly greater than those in Group C (*P *< 0.05). The epidermal SOS values (Figure 3 *bottom*) in Group C samples were higher than those in groups A and B; however, the difference was not statistically significant (*P *> 0.05). Following pepsin treatment, SOS values of both the dermis and epidermis were gradually decreased in all age groups. Moreover, SOS values of both the dermis and epidermis between 0h and 2h following pepsin treatment were significantly different for all age groups (*P *< 0.01).A rapid decrease in the epidermal SOS (*P *< 0.01) after 0.5h was observed only in Group C, but not in groups A and B.

**Table 1.** Comparison of SOS after pepsin among different age groups.

Ret derm	Points	SOS Ave m/s	SD
A–0 h	55	1779.75	72.12
A–0.5 h	55	1745.75	82.46
A–2 h	55	1722.45	78.16
B–0 h	45	1772.85	84.67
B–0.5 h	45	1729.53	64.38
B–2 h	45	1691.94	53.08
C–0 h	55	1733.97	61.69
C–0.5 h	55	1704.28	61.41
C–2 h	55	1666.15	58.82
Pap derm	Points	SOS Ave m/s	SD
A–0 h	25	1645.99	65.19
A–0.5 h	25	1606.52	54.84
A–2 h	25	1575.92	39.49
B–0 h	25	1632.98	61.34
B–0.5 h	25	1595.22	56.55
B–2 h	25	1567.20	56.80
C–0 h	25	1586.22	30.54
C–0.5 h	25	1556.10	36.13
C–2 h	25	1522.43	22.85
Epidermis	Points	SOS Ave m/s	SD
A–0 h	30	1612.9	55.37
A–0.5 h	30	1586.6	64.08
A–2 h	30	1530.87	29.61
B–0 h	30	1593.8	27.57
B–0.5 h	30	1559.9	45.25
B–2 h	30	1518.31	31.91
C–0 h	30	1629.52	61.16
C–0.5 h	30	1582.68	67.02
C–2 h	30	1538.24	27.92
Group	Age Ave ± SD	Gender	
A	39.5 ± 10.5	M4F2	
B	62.6 ± 7.1	M3F3	
C	83.8 ± 5.06	M3F3	

Ret derm, reticular dermis; Pap derm, papillary dermis; A, younger adult group; B, older adult group; C, elderly group; SOS, speed-of-sound; M, male; F, female

### Relationship between epidermal thickness and SOS values

**Figure 4.**
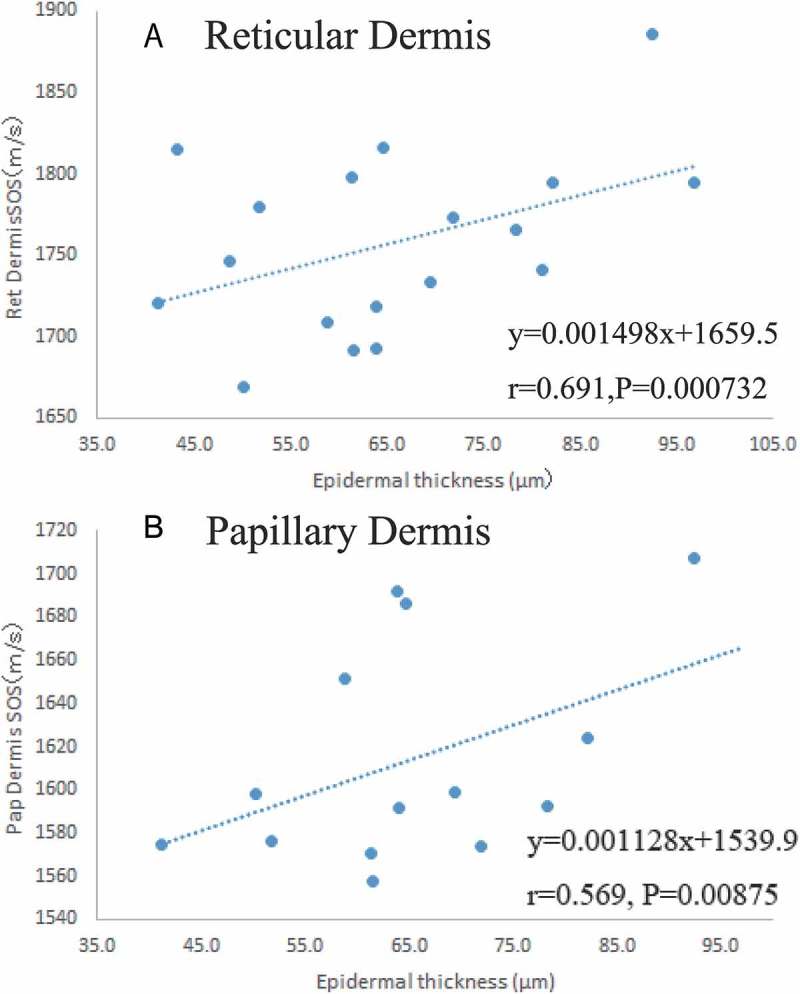
Relationship between epidermal thickness and SOS values. Average SOSs of reticular (a) and papillary (b) dermal SOS were plotted according to each age. Both dermal SOSs had strong and positive correlation with epidermal thickness. SOS, speed of sound.

In the elderly, the epidermis was thinner and the basement membrane was flatter and more conspicuous compared to that in the younger adults. Figure 4(a,b) show the relationship between epidermal thickness and SOS of reticular and papillary dermis, respectively. SOS of reticular and papillary dermis showed a positive correlation with epidermal thickness (*P *= 0.00073, *P *= 0.0087). No significant correlation was observed between SOS and epidermal thickness (*P *= 0.59).

### SOS values in DM and non-DM specimens

**Figure 5.**
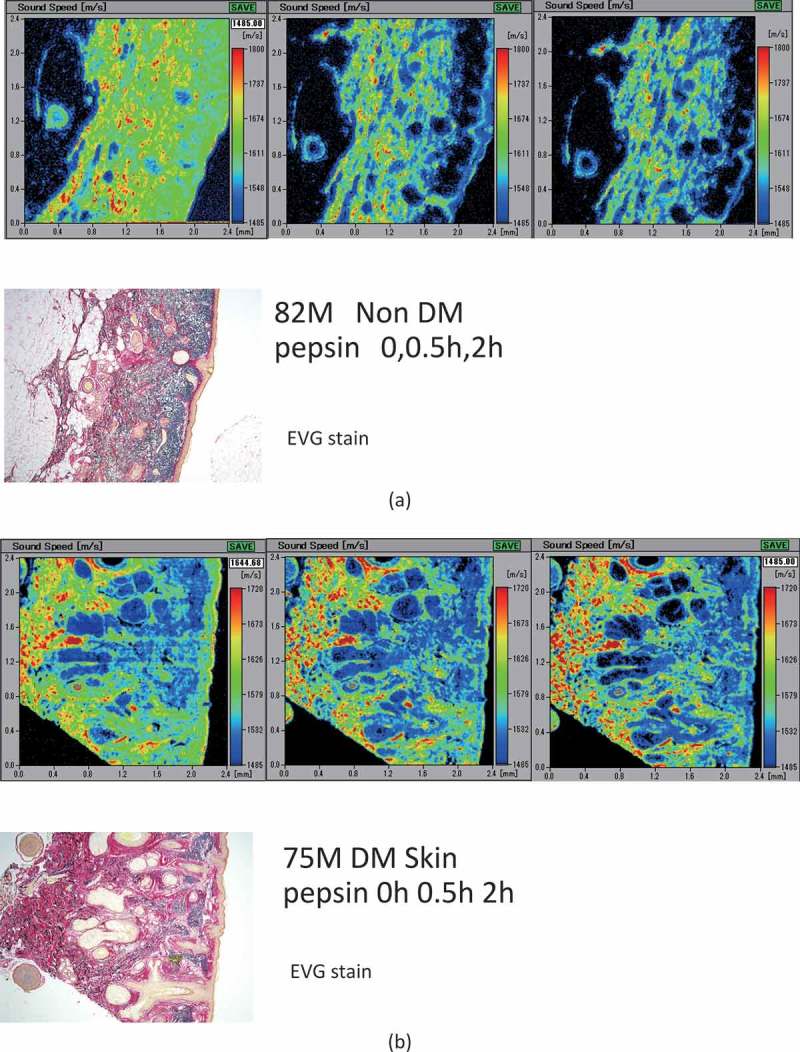
Non-DM (a) and DM (b) skin after pepsin digestion. The dermis and epidermis of non-DM skin (a) showed a rapid reduction in speed of sound (SOS) values after treatment with pepsin. The DM skin (b) showed stable SOS values in the reticular dermis following treatment.

Histologically, the DM and non-DM skin specimens were undistinguishable on LM. The dermis and epidermis of non-DM skin (Figure 5(a)) showed a rapid reduction in SOS values following pepsin treatment, whereas DM skin showed stable SOS values in the reticular dermis after digestion (Figure 5(b)).

**Figure 6.**
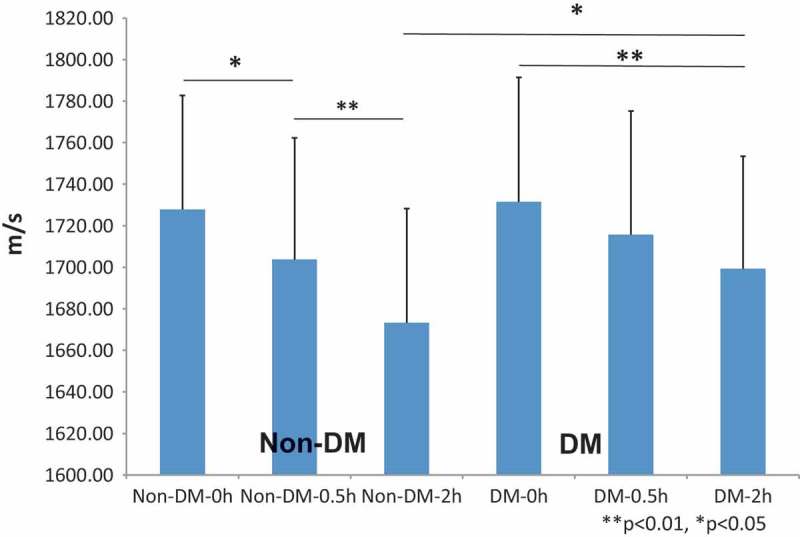
Differences between non-DM and DM skin following pepsin treatment. After pepsin digestion, speed of sound (SOS) values of non-DM skin significantly declined, whereas the values in DM skin decreased more slowly after digestion. Although no significant difference was present at the beginning of digestion, non-DM skin showed a significant reduction in the SOS values 2 h after digestion as compared to DM skin. All SOS values are presented as mean ± standard deviation. **P < 0.01, *P < 0.05, Tukey–Kramer test.

Figure 6 and Table 2 compare SOS values of the reticular dermis between DM and non-DM. Before digestion, no significant difference was found; however, SOS values in non-DM skin showed a significant rapid decrease from 0.5h (*P *< 0.05) following digestion, whereas SOS values in DM skin showed a gradual decrease over the first 2 h after treatment. At 2h after digestion, SOS values in non-DM skin were significantly lower than those in the DM skin (*P *< 0.05).

**Table 2.** Comparison of SOS after pepsin between DM and non-DM patients.

Group	Time	Points	SOS ave m/s	SD
Non-DM, n = 11	0 h	105	1727.8	54.83
	0.5 h	105	1703.7	58.62
	2 h	105	1673.22	55
DM, n = 5	0 h	90	1731.5	59.93
	0.5 h	90	1715.67	59.49
	2 h	85	1699.31	54.03
Group	Age Ave ± SD	Gender		
Non-DM	80.7 ± 7.9	M6F5		
DM	71.4 ± 11.2	M4F1

### Differences between acute and chronic wounds

**Figure 7.**
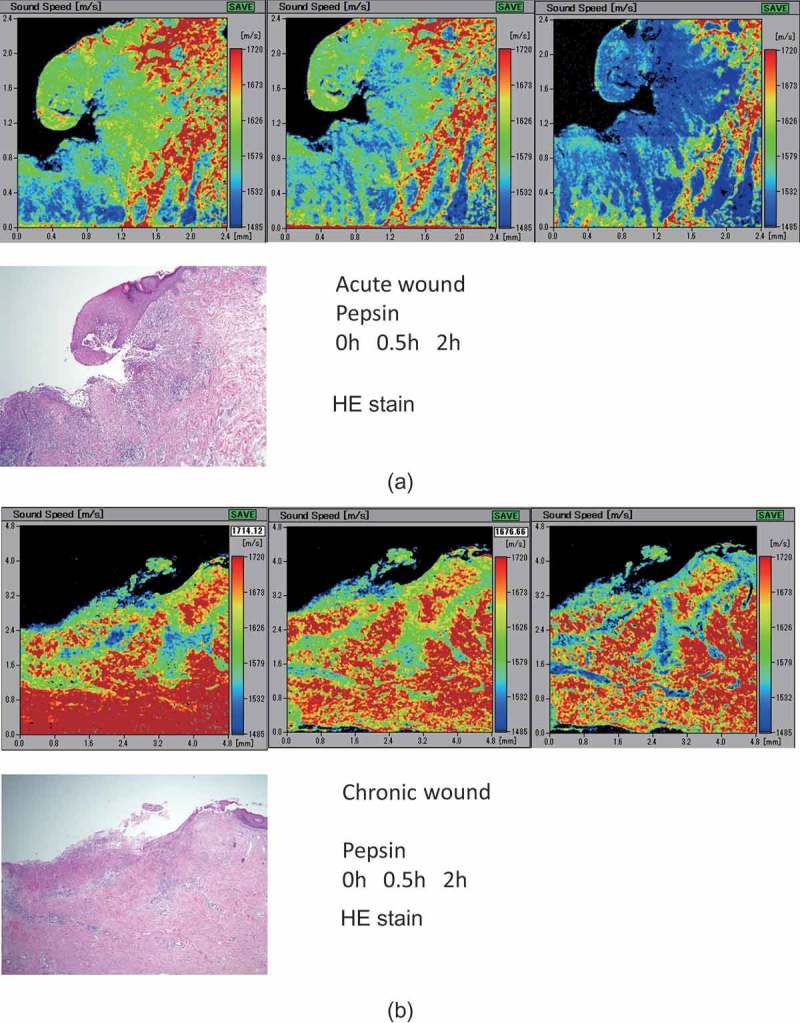
Speed of sound (SOS) images of acute (a) and chronic (b) wounds following pepsin treatment. Acute wounds with an ulcer (left side) showed high SOS values, which rapidly decreased following treatment, whereas the chronic wounds maintained higher SOS values after digestion.

SOS images of acute wounds (Figure7(a)) revealed a substantial reduction in SOS values of ulcer beds composed of fibrous granulation tissue compared to the stably high values of the chronic wounds (Figure7(b)) that consisted of dense collagen. Moreover, SOS images of ulcer beds corresponded to the collagen density observed in the EVG-stained images.

**Figure 8.**
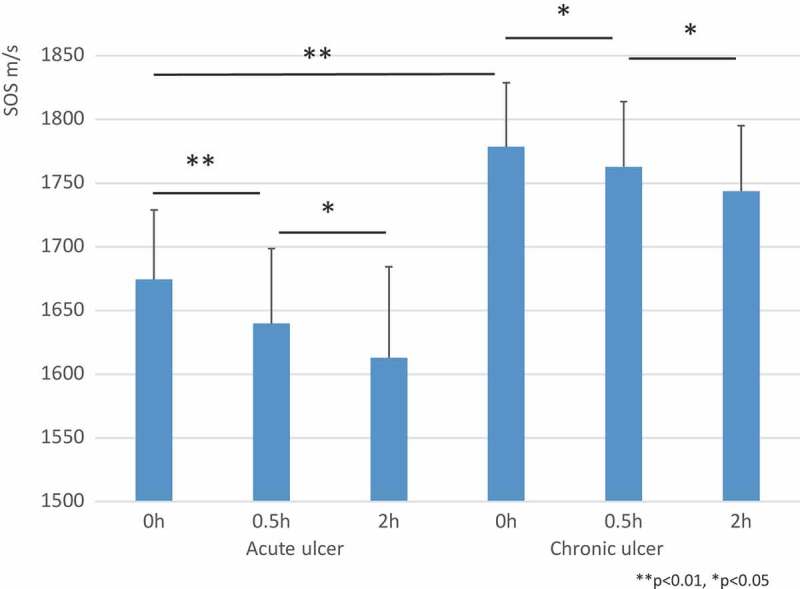
Differences in speed of sound (SOS) values between acute and chronic wounds following pepsin treatment. Chronic wounds had significantly greater SOS values than acute wounds before digestion. Although both the acute and chronic wounds showed a significant reduction in SOS values following treatment, the acute wounds underwent a more rapid reduction at 0.5 h. All SOS values are presented as mean ± standard deviation. **P < 0.01, *P < 0.05, Tukey–Kramer test.

Figure 8 and Table 3 present SOS values of acute and chronic ulcers following pepsin treatment. The SOS values in the acute wounds were significantly lower than those in the chronic wounds (*P *< 0.01). Although both the acute and chronic wounds showed a significant decrease in SOS values after digestion, the acute wounds showed a more rapid reduction.

**Table 3.** Comparison of SOS after pepsin between acute and chronic wounds.

Group, n	Time	Points	SOS ave m/s	SD
Acute, n = 5	0 h	35	1674.44	54.61
	0.5 h	35	1639.77	58.77
	2 h	35	1612.9	71.47
Chronic, n = 4	0 h	40	1778.46	50.3
	0.5 h	40	1762.76	51.07
	2 h	40	1743.74	51.19
Group	Age Ave ± SD	Gender		
Acute	58.4 ± **9.8**	M4F1		
Chronic	58.2 ± 18.2	M3F1

## Discussion

SOS imaging of various skin samples not only showed high-resolution histology comparable to LM but also exhibited mechanical alterations among lesions. Age-related reduction in collagen content in the reticular and papillary dermis was reflected in the declined SOS values in the elderly, which indicated that dermis in the elderly was softer and looser than that in younger adults. Elderly women, in particular, showed significant reduction in stiffness of reticular dermis as compared to that in younger women and elderly men. Compared to the dermis, epithelial SOS values of the elderly tended to be greater than those of younger adults, which indicated greater rigidity and enhanced vulnerability to pepsin digestion.

Skin aging occurs through two distinct processes: intrinsic and extrinsic aging [12,13]. The former is a naturally occurring process, while the latter occurs due to environmental factors (e.g., exposure to ultraviolet light).These aging processes appear in the facial skin. In the elderly, the histological differences are primarily because of atrophy and reduction of most cutaneous components [14–16].In addition, the epidermal changes associated with skin aging include epidermal thinning and flattening of dermal–epidermal junction. Keratinocytes in the epidermis become less adherent to one another. Dermal changes involve alteration and reduction of extracellular matrix (ECM) such as collagen, elastin, and ground substance, which confer tensile strength, resilience (recoil) and hydration, respectively. In chronically sun-exposed skin, an elastotic material composed of elastin, fibronectin, fibrillin, and glycosaminoglycan accumulate in the papillary dermis (solar elastosis). In addition, collagen bundles become thicker and stiffer.

SOS of reticular and papillary dermis showed a strong positive correlation with epidermal thickness. Epidermal thickness indicates dermal mechanical strength. Age-related epidermal thinning [17] particularly in women on the face [18], renders the dermis vulnerable to mechanical damage. Measurement of epidermal thickness can provide an indication of the mechanical weakness of the dermis.

Protease digestion was performed with pepsin. Pepsin has a wider substrate specificity than that of collagenase derived from clostridium, which primarily digests the repeating Gly-X-Y collagen sequence. We initially tried to use this collagenase. The reduction in SOS values, however, was unremarkable, and we replaced it with pepsin for protease digestion. Other matrix-digesting enzymes may show different results.

The remarkable longevity of extracellular matrix (ECM) proteins is believed to predispose them to the risk of chemical modifications. The half-life of ECM proteins is longer than that of intracellular proteins, many of whom have a half-life of hours or days at most. For example, type-1 collagen in human skin has an estimated half-life of 15 years [19]. Elastins in human skin also have marked longevity [20]. The cumulative effect of modification of ECM proteins results in altered functionality, as observed in DM and elderly.

As compared to non-DM skin, DM skin was shown to be more resistant to pepsin degradation in this study. Cross-linking among collagen fibers (e.g., AGEs) increases with age [21–24] and might interfere with pepsin digestion [9,25]. Protease resistance may slow the recovery from inflammation and delay wound repair, as is often observed in DM patients [26].

DM is considered as a modern epidemic disease that affects about 425 million people globally, and half of cases are estimated to be currently undiagnosed [27]. There are many proposed pathological mechanisms of skin involvement in DM, which include abnormal carbohydrate metabolism [28]. Diabetic skin shows increased expression of lysyl oxidase (LOX) and higher cross-linked collagen [29]. Mechanical properties of traction force and tensile strength are increased in diabetic skin, as compared to intact/well-organized collagen fibrils in non-DM skin.

In the wound repair lesions, SAM was able to discriminate between acute and chronic wound samples because fresh wounds were much softer than chronic wounds and were more sensitive to pepsin digestion. We have previously reported the use of this protease method to evaluate acute and older myocardial infarction, as well as experimental mouse ulcer repair [9]. By assessing the resistance to protease digestion, SAM may be used to estimate the duration of fibrosis. Collagen fibers in younger adults, which appeared in the granulation tissues following infarction or inflammation, were vulnerable to protease digestion; however, chronic fibrotic tissues (e.g., chronic infarction or scar tissues) showed great resistance to protease degradation.

Wound healing process consists of four sequential phases, blood clotting (hemostasis), inflammation, tissue growth (proliferation), and tissue remodeling. The healing process is susceptible to interruption, which may lead to nonhealing chronic wounds. Evaluation of the healing phase from histological sections may benefit patient care, as the degree of fibrosis corresponds to rigidity, which is reflected in the SOS values obtained on SAM.

Several limitations of this study should be acknowledged. First, the number of samples used to compare the results was not sufficient. Use of a greater number of samples may have led to higher *p* values. We attempted to gather more samples to compare skin SOS. However, excisional facial skin samples containing enough normal portions were limited because biopsy samples were too small to contain normal epidermal and dermal portions [18]. Second, we used FFPE samples and not the fresh frozen samples. While FFPE sections of skin had higher SOS values as compared to those of unfixed sections, they reflected the original values of fresh tissue. Our previous study showed that SOS values of FFPE tissues were stable after fixation for durations ranging from 24 h to 3 months. Third, our results may have been affected by measurement bias. To minimize the effect of measurement errors, we obtained at least three SOS images from the same location and compared these with the LM images. SOS data were collected from at least five different points from each image.

Age-related changes in other organs (e.g., musculoskeletal and cardiovascular system) may be evaluated using these methods since changes in collagen turnover in these systems may cause age-related mechanical transformation. Protein glycation and formation of AGEs play an important role in the pathogenesis of diabetic complications such as retinopathy, nephropathy, neuropathy, and cardiomyopathy. AGEs form intra- and extracellular cross-linkages not only with proteins, but also with some other endogenous molecules, including lipids and nucleic acids, to contribute to the development of diabetic complications [30]. As wound healing and its dysregulation via fibrosis occur in all tissues, early detection of this process may help prevent disease progression. SAM, which allows for calculation of SOS through tissues, may represent a novel means to evaluate various forms of organ damage.

In conclusion, SOS imaging of routine skin sections cannot only afford fine-resolution histology but also mechanical properties to evaluate aging, DM state, and the stage of wound repair.
