# Supplementary material for: Evaluation of aging, diabetes mellitus, and skin wounds by scanning acoustic microscopy with protease digestion
Source: Pathobiol Aging Age Relat Dis. 2018 Sep 6;8(1):1516072. doi: 10.1080/20010001.2018.1516072 (PMC6136385; doi:10.1080/20010001.2018.1516072)
Supplement: Supplemental Material [file ZPBA_A_1516072_SM8968.docx]

**Supplemental figures**


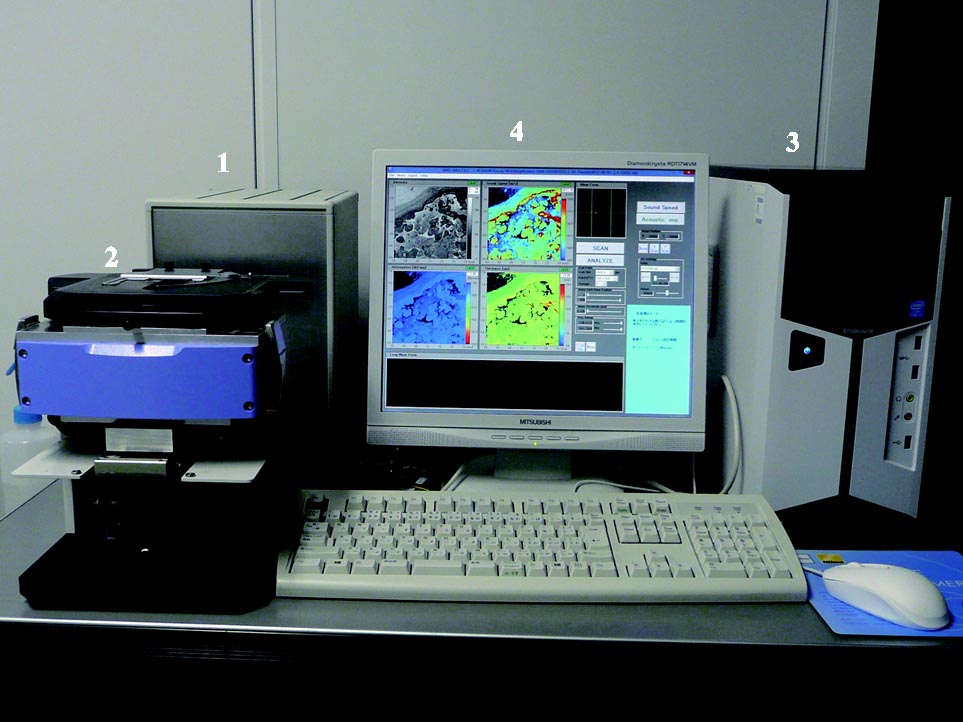


Supplemental figure 1. Appearance of scanning acoustic microscope systems. 1. Signal processor, 2. mechanical scanner with transducer, 3. system control PC, and 4. display.


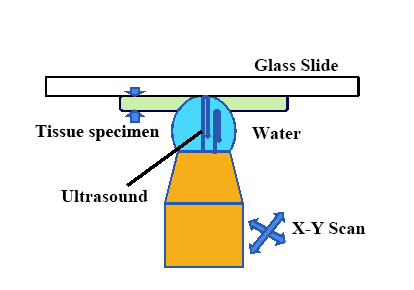


Supplemental figure 2. Principle of SAM. Ultrasonic waves from the transducer reflect off both the glass slide and the sample section before returning to the transducer. The waves pass through 10-µm sample sections with different ultrasonic properties. The transducer automatically scans the section to calculate the speed of sound (SOS) through each area. The section is placed upside down on the transducer, and distilled water is applied between the transducer and the section as a coupling fluid. The control SOS through water is 1,495 m/s.


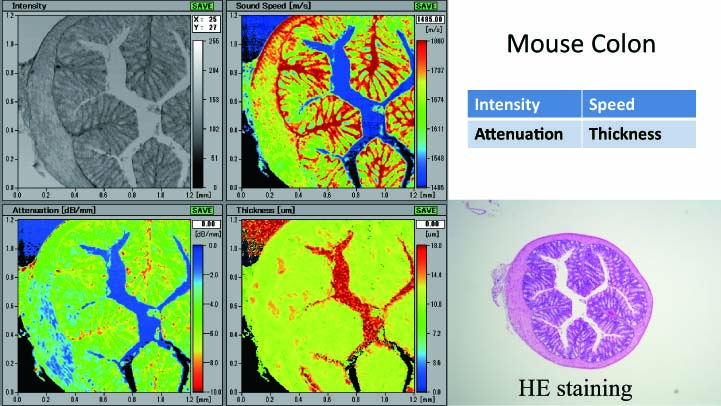


Supplemental figure 3. Histology obtained by SAM. After mechanical X–Y scanning, the SOS is calculated from each point on the section and plotted on the screen to create two-dimensional, color-coded images. Data of intensity, attenuation of sound, and thickness are also obtained from each point to make histological images. Each image are comparable with light microscopic one.
